# Ultrasound-Guided Selective Glossopharyngeal and Maxillary Nerve Blocks for Analgesia After Tonsillectomy: A Pilot Study

**DOI:** 10.7759/cureus.68672

**Published:** 2024-09-04

**Authors:** Kana Inoue, Yuki Kojima, Takahito Kuga, Kazuya Hirabayashi

**Affiliations:** 1 Anesthesiology, Asahi General Hospital, Asahi, JPN

**Keywords:** postoperative analgesia, ultrasound-guided, tonsillectomy, maxillary nerve block, glossopharyngeal nerve block

## Abstract

Purpose

Postoperative pain management methods for tonsillectomy commonly include the use of opioids, non-steroidal anti-inflammatory drugs, and acetaminophen. However, some patients report pain despite the use of these medications. In recent years, ultrasound-guided selective glossopharyngeal nerve block (UGSGNB) and ultrasound-guided maxillary nerve block (UGMNB) have been reported to be effective for analgesia post-tonsillectomy. We retrospectively analyzed the effects of UGSGNB and UGMNB in the perioperative management of patients who underwent tonsillectomy under general anesthesia.

Methods

This retrospective study evaluated adults (18-61 years old) who had received general anesthesia for tonsillectomy. The control group comprised 25 patients who received general anesthesia using the standard protocol, and the nerve block group comprised 10 patients who also received additional UGGNB and UGMNB.

Results

While these nerve blocks may have contributed to improving the postoperative food intake, they did not reduce the frequency of postoperative analgesia used. Improved dietary intake after UGSGNB and UGMNB could be advantageous for postoperative recovery.

Conclusion

Further research with a larger number of cases and prospective intervention studies are necessary to determine the effects of combining UGSGNB and UGMNB for post-tonsillectomy analgesia.

## Introduction

Opioids, nonsteroidal anti-inflammatory drugs (NSAIDs), and acetaminophen are commonly used for pain management after tonsillectomies [1]. However, opioids may be unsuitable for controlling postoperative pain in some cases, particularly in patients with tonsillar hypertrophy-associated obstructive sleep apnea. Increased sensitivity to opioids due to obstructive sleep apnea may complicate postoperative management and contribute to postoperative nausea and vomiting [2,3]. In recent years, glossopharyngeal nerve block (GNB) has been widely used for pain management [4-7]. Ultrasound-guided selective glossopharyngeal nerve block (UGSGNB) is a modified version of GNB that addresses the limitations of traditional GNB [4]. Kojima and Oiwa [8] reported the efficacy of UGSGNB in treating severe gag reflexes. Additionally, the concurrent use of ultrasound-guided maxillary nerve block (UGMNB) may be effective for managing post-tonsillectomy pain [9-11]. We hypothesized that UGSGNB and UGMNB could serve as effective postoperative analgesia methods in patients undergoing bilateral tonsillectomy. Herein, we retrospectively analyzed the effects of UGSGNB and UGMNB on the perioperative management of tonsillectomy under general anesthesia.

## Materials and methods

This retrospective pilot study evaluated adults (aged 18-61 years) who underwent tonsillectomy under general anesthesia at the Department of Anesthesiology, Asahi General Hospital (Asahi City, Chiba, Japan) between January 2023 and February 2024. This study is registered in a publicly accessible database (UMIN Clinical Trials Registry ID: UMIN000054183). All patients provided informed consent for the treatment, and the study protocol was approved by the Asahi General Hospital Review Board (Approval No: 2024031914). 

The patients were divided into the control (n = 25) and nerve block (n = 10) groups. The control group received general anesthesia using the standard protocol, whereas the nerve block group received additional UGSGNB and UGMNB. Patients with surgical sites that extended beyond the pharynx were excluded. 

General anesthesia was induced using propofol, rocuronium, fentanyl, and remifentanil as needed, followed by tracheal intubation and mechanical ventilation. Anesthesia was maintained using remifentanil, propofol, desflurane, or sevoflurane with an oxygen/air mixture. UGSGNB and UGMNB were performed after inducing general anesthesia. Ropivacaine was administered to each side at a dose of 0.2-0.25% (2-3 mL) for UGSGNB and 0.375% (5-6 mL) for UGMNB. We used acetaminophen or flurbiprofen during surgery. After recovery from general anesthesia, the patients were transferred to a surgical ward and received standard analgesic treatment with acetaminophen or NSAIDs. 

Information on the total opioid use, non-opioid analgesic use, and dietary intake were obtained from patient medical records. Complications associated with UGMNB and UGSGNB were also recorded. Food intake was evaluated on a 5-point Likert scale (0 = no food intake; 5 = complete meals) at each meal by the ward nurse. Data analyses were performed using the ORIGIN® (Origin Lab Corporation, Northampton, MA, USA) and R (The R Foundation for Statistical Computing, Vienna, Austria) packages. Continuous variables are presented as the median and interquartile range (IQR), and categorical variables are presented as numbers (percentages). Intergroup differences were evaluated using the Wilcoxon rank sum test. All reported P-values are two-sided, with statistical significance set at P < 0.05.

## Results

No significant differences were observed in age, height, weight, and body mass index between the nerve block and control groups (Table 1). However, there were significant differences in dietary intake on postoperative day (POD) 1 and the use of acetaminophen and NSAIDs during surgery (P < 0.05). Dietary intake on POD 2 and 3 was higher in the nerve block group than in the control group. We observed no differences in the use of postoperative analgesics between the groups. Moreover, there were no complications associated with UGMNB or UGSGNB, such as hematoma, puncture of internal organs or vessels, cardiac or neurological side effects, infection, or allergic reactions.

**Table 1:** Patient characteristics and results in the control and nerve block groups There were significant differences in dietary intake on postoperative day (POD) 1 and the use of acetaminophen and NSAIDs during surgery. Dietary intake on POD 2 and 3 was higher in the nerve block group than in the control group. Continuous variables are presented as the median and interquartile range (IQR). NB; nerve block; BMI: body mass index; POD: postoperative day.

Variable	NB group (n = 10)	Control group (n = 25)	P value
Sex	Female: 5 Male: 5	Female: 15 Male: 10	0.58
Age (years)	28.5 (19, 61)	26.0 (18, 48)	0.41
Height (cm)	163 (150, 181)	161 (147, 177)	0.91
Weight (kg)	57 (43, 85)	62 (45, 102)	0.41
BMI (kg/m^2^)	23 (16, 31)	23 (17, 32)	0.47
During operation			
Dose of remifentanil (mg)	0.9 (0.7, 2)	1 (0.4, 2.3)	0.65
Dose of fentanyl (µg)	150 (100, 300)	100 (0, 300)	0.34
Other analgesics use	Flurbiprofen: 0 (0%)	Flurbiprofen: 13 (52%)	< 0.01*
	Acetaminofen: 6 (60%)	Acetaminofen: 24 (96%)	0.01*
Dietary intake			
POD 1	15 (3, 15)	11 (0, 15)	0.04*
POD 2	15 (0, 15)	11 (1, 15)	0.06
POD 3	15 (2, 15)	10 (1, 15)	0.07
Postoperateive amounts of analgesics			
POD 1	2 (0, 4)	2 (0, 5)	0.38
POD 2	2 (0, 3)	2 (0, 5)	0.21
POD 3	2 (0, 4)	2 (0, 4)	0.95

## Discussion

In this study, we analyzed the effects of UGSGNB and UGMNB on the perioperative management of tonsillectomy under general anesthesia. Our findings suggest that the effects of UGSGNB and UGMNB may enhance postoperative dietary intake after tonsillectomy by providing effective anesthesia, which facilitates eating. Increased dietary intake significantly contributes to postoperative recovery. Considering the concept of “Enhanced Recovery After Surgery,” implementing UGSGNB and UGMNB could be highly effective for tonsillectomy. Additionally, the use of nerve blocks may help reduce opioid consumption.

However, it is essential to consider that dietary intake varies among individuals and cannot be precisely measured. Notably, there were no significant differences in the postoperative use of analgesics, such as acetaminophen and NSAIDs, between the nerve block and control groups. The dose of remifentanil and fentanyl administration during operation were also similar between both groups. Recurrent severe inflammation increases tonsil adhesion before surgery. In cases that demonstrate invasion into areas beyond the effective range of the glossopharyngeal and maxillary nerve blocks, the desired effects may not be achieved [11, 12]. Therefore, there is a need to reassess the approach to using nerve blocks in tonsillectomies, and the techniques and approaches should be tailored to the clinical situation. Depending on the case, the use of opioids, acetaminophen, or flurbiprofen may be necessary. Although there are no studies addressing the complications of UGMNB, there have been reports on the complications of UGMNB. Kojima et al. reported no complications in 167 cases [13], suggesting that UGMNB may be safer and simpler to perform than conventional techniques [9]. As the ultrasound-guided approach allows for real-time visualization of anatomical structures, it may reduce the risk of complications [13].

Our study has a few limitations. First, the small sample size (n = 35) limits the generalizability of our findings and ability to identify statistically significant differences between the groups. Second, retrospective studies are inherently susceptible to bias as data are collected from existing medical records rather than being prospectively collected under controlled conditions. Additionally, this study did not include pediatric cases. While UGMNB has been applied to children [14-16], it is necessary to consider whether UGSGNB can be safely applied in pediatric populations. The optimal amount of local anesthetic for each nerve block should be investigated in a cadaver study. In summary, while our findings are encouraging, future prospective studies with larger sample sizes are necessary to confirm our findings on the use of UGSGNB and UGMNB for pain management.

## Conclusions

This report describes the effects of UGSGNB and UGMNB on post-tonsillectomy pain management. Our results identified an increase in postoperative dietary intake after UGSGNB and UGMNB. These nerve blocks can effectively provide analgesia without intraoperative NSAIDs and acetaminophen. However, further clinical studies in larger cohorts are required to validate our findings.
